# A simple stochastic model describing the evolution of genomic GC content in asexually reproducing organisms

**DOI:** 10.1038/s41598-022-21709-z

**Published:** 2022-11-03

**Authors:** Jon Bohlin

**Affiliations:** 1 Division of Infection Control, Department of Methods Development and Analysis, Norwegian Institute of Public Health, Oslo, Norway; 2 Centre for Fertility and Health, Norwegian Institute of Public Health, P.O. Box 4404, Lovisenberggata 8, 0403 Oslo, Norway

**Keywords:** Genome evolution, Statistical methods, Evolutionary theory

## Abstract

A genome’s nucleotide composition can usually be summarized with (G)uanine + (C)ytosine (GC) or (A)denine + (T)hymine (AT) frequencies as GC% = 100% − AT%. Genomic AT/GC content has been linked to environment and selective processes in asexually reproducing organisms. A model is presented relating the evolution of genomic GC content over time to AT $\rightarrow$ GC and GC $\rightarrow$ AT mutation rates. By employing Itô calculus it is shown that if mutation rates are subject to random perturbations, that can vary over time, several implications follow. In particular, an extra Brownian motion term appears influencing genomic nucleotide variability; the greater the random perturbations the more genomic nucleotide variability. This can have several interpretations depending on the context. For instance, reducing the influence of the random perturbations on the AT/GC mutation rates and thus genomic nucleotide variability, to limit fitness decreasing and deleterious mutations, will likely suggest channeling of resources. On the other hand, increased genomic nucleotide diversity may be beneficial in variable environments. In asexually reproducing organisms, the Brownian motion term can be considered to be inversely reflective of the selective pressures an organism is subjected to at the molecular level. The presented model is a generalization of a previous model, limited to microbial symbionts, to all asexually reproducing, non-recombining organisms. Last, a connection between the presented model and the classical Luria–Delbrück mutation model is presented in an Itô calculus setting.

## Introduction

The hereditary material of living organisms consist of double stranded deoxyribonucleic acid (DNA) molecules^1^. The building blocks of the DNA molecule are the nucleotides Adenine (A), Guanine (G), Cytosine (C) and Thymine (T)^1^. Across each strand Gs pair with Cs and Ts with As only^1^. Within each strand the different nucleotides are stacked in no specific order connected via a sugar backbone^1^. Virus can have genomes consisting of single or double stranded DNA or ribonucleic acid (RNA)^2^. RNA is similar to DNA but is more often single stranded and T is substituted for Uracil (U)^3^. Genomes consisting of double stranded DNA have approximately as many As as Ts and Gs as Cs on each strand^4^. These relations were first observed by Erwin Chargaff^4^ and are therefore referred to as one of Chargaff’s parity laws. Base composition in genomes with double stranded DNA can therefore be analyzed using either AT- or GC content as GC% = 100% − AT%.

Base-pairing across the two DNA strands consists of three hydrogen bonds for G and C and two for A and T^1^. More energy is in general required to stack and melt G and C bindings as compared to A and T^5^. Methylation of C, by the addition of a methyl group, often results in deamination of C transforming it to T^6^. Deamination of methylated Cs is therefore implicated in an AT mutational bias observed for bacteria and archaea (prokaryotes)^7^. Relaxation of selective pressures are hypothesized to increase AT content in microbial genomes due to the failure of repair enzymes to remove methylated cytosines^8^. Indeed, both modelling and empirical investigations points to an approximately 2:1 relationship of respectively GC $\rightarrow$ AT and AT $\rightarrow$ GC mutation rates in prokaryotes^7,9^. It should be noted that AT $\rightarrow$ GC mutations (and vice versa) are in the present work taken to mean all possible combinations of A and T to G and C (G and C to A and T). DNA methylation also occurs in organisms with a cell nucleus (eukaryotes) and it is therefore reasonable to expect an AT mutational bias for these organisms as well^9^. However, as many larger multi-cellular organisms reproduce sexually homologous recombination may obscure any such mutational AT bias observed for non-recombining asexually reproducing organisms^6^. Homologous recombination seems to increase GC content in eukaryotes as a consequence of a process referred to as GC-biased gene conversion^10^. Identifying AT/GC mutational biases is therefore difficult in sexually reproducing organisms and thus not considered in the present study. For larger multi-cellular organisms the GC content is fairly stable between species although there can be local genomic differences, for instance CpG islands, which are usually not present in prokaryotes^6^. For prokaryotes, within-species genomic GC content is stable while GC content between different species can vary substantially, from 13.5% GC to 75% GC^11^. This variation in genomic GC content appears to have both an environmental component as well as a phylogenetic one^12,13^. Environmental influence on genomic GC content in prokaryotes appears to be mediated, at least to some extent, as selective pressures^14,15^. In this respect, it is of interest to note that large drops in genomic GC content is wide-spread in prokaryotes^7^ while examples of substantial increase is practically non-existent as of now^16^, although smaller increases are documented^13,15,17,18^. Examination of AT- and GC mutation rates in prokaryotes points to an AT bias also in recombining microbes while AT $\rightarrow$ GC substitutions are more likely to be retained^9,19^.

A previous study^11^ modelling the evolution of genomic GC content in microbes living in a stable symbiotic relationship with an eukaryotic host, usually insects, suggested that AT $\rightarrow$ GC mutation rates, and vice versa, may determine the symbiotic species fate early on in the organism’s history provided mutation rates are approximately constant. Microbial symbionts often live in low density populations, are unable to perform homologous recombination and lack many DNA repair genes implying that mutations accumulate, often in a clock-like manner^20^. If the selective pressures are not strong enough to purge deleterious and fitness decreasing mutations from the symbiont’s genome, it will decay^21^. This process is known as Muller’s ratchet^22^. For clonal, non-recombining symbionts, the evolutionary process of Muller’s ratchet will always incur, the only question is when^22^.

Since microbial symbionts do not recombine and live in a stable environment with their eukaryotic host it makes sense to model the random perturbations of their AT- and GC mutation rates as a Gaussian white noise multiplied by a constant, or a fixed parameter, to be estimated, as has previously been done^11^. For non-recombining, asexually reproducing organisms in general, subjected to differing selective pressures over time, modeling AT/GC mutation rate perturbations as a Gaussaian white noise multiplied with a constant would be too simplistic. It is more natural to model random perturbations of AT/GC mutation rates in such organisms as a Gaussian white noise multiplied by a function varying with respect to time, as this will allow the model to account for varying selective pressures. As such, the purpose of the present work is to model the evolution of genomic GC content as a consequence of AT $\rightarrow$ GC, as well as GC $\rightarrow$ AT, mutation rates subject to random perturbations *c*(*t*) over time *t* for all non-recombining, Chargaff parity law compliant organisms, regardless of which kingdom they belong to, and explore the evolutionary implications. A connection between the derived model and the classical Luria-Delbruck mutation model^23^ is also established.

## Methods

### The mathematical model

The model presented in this study is an extension of models from previous work^9,11,19^. A brief overview and elaboration of these models is included in the Supplementary Appendix. In particular, the present work is an extension of a model describing the evolution of genomic GC content as a consequence of AT/GC mutation rates with random perturbations for microbial symbionts^11^. As there are many similarities between present- and the previous work on microbial symbionts^11^ only the steps separating these models and details necessary for a complete comprehension are included.

First, $F_t(\omega )$ represents genomic GC content at time *t* for all trajectories $\omega \in \Omega$ (for details see^11,19^) such that:

1 $$\begin{aligned} F_{t+\Delta t} (\omega ) - F_t (\omega ) = \alpha F_t (\omega )\Delta t+\beta (1-F_t (\omega ))\Delta t. \end{aligned}$$

That is, the change in genomic GC content $F_{t+\Delta t} (\omega ) - F_t (\omega )$ during time $\Delta t$, for trajectory $\omega \in \Omega$, is a fraction multiplied with genomic GC- and AT content, respectively described by $\alpha F_t (\omega )\Delta t$ and $(\beta (1-F_t (\omega )))\Delta t$. Somewhat inaccurately this will be interpreted as the GC content of single nucleotide polymorphisms (SNPs/variable sites) in a species during time $\Delta t$. It will be shown later that there is a natural way of accurately extracting SNP GC/AT content from Eq. (1). If it is assumed that $\Delta t\rightarrow 0$ Eq. (1) can be written as a differential equation:

2 $$\begin{aligned} \frac{dF_t (\omega )}{dt} = \alpha F_t (\omega ) + \beta (1-F_t (\omega )). \end{aligned}$$

The AT $\rightarrow$ GC and GC $\rightarrow$ AT mutation rates, designated as $\alpha$ and $\beta$ respectively, are subject to random perturbations $W_t(\omega )$ that can vary with time multiplied by a function *c*(*t*), i.e. $\alpha =a+c(t)W_t (\omega )$ and $\beta =b+c(t)W_t (\omega )$. It is therefore assumed that *c*(*t*) is a measurable function and that $W_t (\omega )$ is a Gaussian white noise^24^ with respect to all trajectories $\omega$ from the set $\Omega$. Furthermore, Eq. (2) belongs to the probability space $(\Omega , \mathcal {F} _t, P)$ while *c*(*t*) is an element of the measure space $(\mathbb {R}^+,\mathcal {G}, dt)$. $\mathcal {F}_t$ is the filtration of $\Omega$ with respect to each time $t\in \mathbb {R^+}$ (i.e. $[0,\infty )$ of which $\mathcal {G}$ is a Borel algebra and *dt* the corresponding Lebesgue measure), and *P* is a probability (Lebesgue) measure on the space of trajectories $\Omega$. The filtration $\mathcal {F} _t$ is interpreted as the evolutionary history of trajectories $\omega$ up to time *t*. Some further re-arrangements gives:

$$\begin{aligned} \frac{dF_t (\omega )}{dt}= & {} (a+c(t)W_t (\omega )) F_t (\omega ) + (b+c(t)W_t (\omega )) (1-F_t (\omega )) \\= & {} \,aF_t (\omega )+c(t)W_t(\omega )F_t(\omega )+\\&+\,b(1-F_t (\omega ))+c(t)W_t (\omega ) (1-F_t (\omega ))\\= & {} \,aF_t (\omega ) + b(1-F_t (\omega ))+c(t)W_t (\omega ). \end{aligned}$$

The equation:

3 $$\begin{aligned} \frac{dF_t (\omega )}{dt} =aF_t (\omega ) + b(1-F_t (\omega ))+c(t)W_t (\omega ), \end{aligned}$$

can be written as a differential form as was explained in the previous study^11^:

4 $$\begin{aligned} dF_t (\omega ) =(aF_t (\omega ) + b(1-F_t (\omega )))dt+c(t)dB_t (\omega ). \end{aligned}$$

Recall that the white noise process $dW_t$ is often interpreted as $\frac{dB_t}{dt}$ so that $dB_t=dW_t dt$. If $c(t)=c$, where *c* is a constant, Eq. (4) will coincide with the model for microbial symbionts^11^ and so is a generalization of that model. The Brownian motion term can additionally be a *c*-scaled Brownian motion which was previously also shown^11^ to be a Brownian motion there termed $\hat{B_t}$. Using the Itô formula^24^:

5 $$\begin{aligned} dY_t (\omega ) =\frac{\partial g}{\partial t} (t,F_t(\omega ))dt+\frac{\partial g}{\partial t}(t, F_t(\omega ))dF_t(\omega )+\frac{1}{2} \frac{\partial ^2g}{\partial x^2} (t,F_t (\omega ))(dF_t (\omega ))^2. \end{aligned}$$

Equation (4) can be given an explicit solution through the integrating factor $g(t,F_t (\omega ))=Y_t (\omega )=e^{(-(a-b)t)} F_t (\omega )$.

Because $\frac{\partial ^2 g}{\partial x^2}(t,x)=0$ (see^24^ for details), the last term of Eq. (5) is equal to zero. As a result,

$$\begin{aligned} dY_t (\omega )= & {} \frac{\partial g}{\partial t} (t,F_t(\omega ))dt+\frac{\partial g}{\partial x}(t, F_t(\omega ))dF_t(\omega ) \\= & {} -(a-b)e^{(-(a-b)t)} F_t(\omega )dt+e^{(-(a-b)t)}dF_t (\omega ) \\= & {} -(a-b)e^{(-(a-b)t)} F_t(\omega )dt+ \\&+\,e^{(-(a-b)t)}(((a-b)F_t (\omega ) +b)dt+c(t)dB_t) \\= & {} be^{(-(a-b)t)}dt+c(t)e^{(-(a-b)t)}dB_t. \end{aligned}$$

$F_t(\omega )$ can then be given an explicit formula:

$$\begin{aligned} d(e^{(-(a-b)t)} F_t (\omega ))=be^{(-(a-b)t)}dt+c(t)e^{(-(a-b)t)}dB_t, \end{aligned}$$

which, by assuming $s\in [0,t]$, gives

$$\begin{aligned} e^{(-(a-b)t)} F_t (\omega )-F_0(\omega )=\int _0 ^t be^{(-(a-b)s)}ds + \int _0 ^t c(s)e^{(-(a-b)s)} dB_s, \end{aligned}$$

and therefore

6 $$\begin{aligned} F_t (\omega )=F_0(\omega ) e^{(a-b)t}+\int _0 ^t be^{(a-b)(t-s)}ds + \int _0 ^t c(s)e^{(a-b)(t-s)}dB_s. \end{aligned}$$

An analogous argument to the previous study^11^ gives an explicit formula for the finite variation term $\int _0 ^t be^{(a-b)(t-s)}ds$. The quadratic variation Brownian motion term $\int _0 ^t c(s)e^{(a-b)(t-s)}dB_s$ however must be approximated numerically, not least due to the unspecified measurable function *c*(*t*):

7 $$\begin{aligned} F_t (\omega )=-\frac{b}{(a-b)}+(F_0(\omega ) +\frac{b}{(a-b)})e^{(a-b)t}+\int _0 ^t c(s)e^{(a-b)(t-s)}dB_s, \end{aligned}$$

which is subject to the constraints $t\in [0,\infty )$ and $0<F_t (\omega )<1$. The integration constant $c_0$ is just included in $F_0$. It should be noted that for $F_0=0$,

8 $$\begin{aligned} \mathbb {E}(F_t(\omega ))=\frac{b}{(a-b)}\bigg (e^{(a-b)t}-1\bigg ). \end{aligned}$$

Since the Brownian motion term vanishes as a consequence of the expectation operator $\mathbb {E}$ (see p. 30 of Ref.^24^), the solution to (7) when $t=x$ is exactly the model for SNP GC content with respect to core genome GC content *x* described previously^9,19^, a brief elaboration is also included in the Supplementary Appendix. Moreover, this means that it is not necessary to calculate the Brownian motion term when estimating the AT- and GC mutation rate parameters *a* and *b*.

The variance is given by $\mathrm {Var}(F_t (\omega ))=\mathbb {E}((F_t(\omega )-\mathbb {E}(F_t(\omega ))^2)$, which can be solved by setting:

$$\begin{aligned} A:=F_0(\omega ) e^{(a-b)t}+\frac{b}{(a-b)}\bigg (e^{(a-b)t}-1\bigg ), \end{aligned}$$

and

$$\begin{aligned} B:=\int _0 ^t c(s)e^{(a-b)(t-s)}dB_s, \end{aligned}$$

which gives:

$$\begin{aligned} \mathrm {Var}(F_t (\omega ))= &\ {} \mathbb {E}\bigg ((F_t(\omega )\bigg )-\mathbb {E}\bigg (F_t(\omega )\bigg )^2 \\= &\ {} \mathbb {E}\bigg ((A+B)^2-2(A+B)A+A^2\bigg ) \\= &\ {} \mathbb {E}\bigg (A^2+2AB+B^2-2A^2-2AB+A^2\bigg ) \\= &\ {} \mathbb {E}(B^2)=\mathbb {E}\bigg (\bigg (\int _0 ^t c(s)e^{(a-b)(t-s)}dB_s\bigg )^2\bigg ). \end{aligned}$$

Applying the Itô isometry (see p. 26^24^):

9 $$\begin{aligned} \mathbb {E}\bigg (\bigg (\int _0 ^t c(s)e^{(a-b)(t-s)}dB_s\bigg )^2\bigg )=\mathbb {E}\bigg (\int _0 ^t \bigg (c(s)e^{(a-b)(t-s)}\bigg )^2 ds\bigg ) =\int _0 ^t c(s)^2 e^{2(a-b)(t-s)} ds. \end{aligned}$$

The formula cannot be given an analytic representation due to the unspecified function *c*(*t*) but it is clear that the integral $\int _0 ^t c(s)^2 e^{2(a-b)(t-s)} ds \rightarrow \infty$ as *c*(*s*) increases for $s\rightarrow t$. As was argued in the previous study on microbial symbionts^11^, it will be assumed henceforth that $(a-b)<0$ where *a* and *b* are respectively the AT$\rightarrow$GC and GC$\rightarrow$AT mutation rate parameters that can be estimated (see^9,19^). The previous study^11^ also showed that *a* and *b* could be considered as unspecified measurable functions. However, as constant mutation rates are not uncommon^25^ *a* and *b* will henceforth be regarded as constants, or parameters to be estimated, to avoid unnecessary complication of interpretation, formulation and derivation of the model.

### The Brownian motion term

The term:

10 $$\begin{aligned} \int _0 ^t c(s)e^{(a-b)(t-s)}dB_s, \end{aligned}$$

depends on the parameters *a* and *b* as well as on the duration of the time period. Since it is assumed that $(a-b)< 0$, $c(s)e^{(a-b)(t-s)}\rightarrow c(s)$ for $s\rightarrow t$. The term can be written as:

11 $$\begin{aligned} \int _0 ^t c(s)e^{(a-b)(t-s)}dB_s=\lim _{\Delta s_i \rightarrow 0} \sum _{s_0} ^{s_N} c(s_i)e^{(a-b)(t-s_i)}(W_{s_{i+1}} (\omega ) - W_{s_i}(\omega ))\Delta s_i, \end{aligned}$$

where $W_s (\omega )$ is scaled white noise with mean $\mu =0$, variance $\sigma ^2 =1$ (see^19^), $\Delta s_i =s_{i+1}-s_i$, and $s_0=0,\ldots ,s_i=t_i,\ldots ,s_N=t$. The right hand side term of Eq. (11) can be calculated manually by inserting values for each value $s_i$ and mutation parameters *a* and *b*. Each Gaussian white noise $W_{s_i}(\omega )$ can be sampled from a normal distribution.

### The Girsanov transform

The Girsanov transform implies that Eq. (7), if an appropriate transform exists, can be considered as a Brownian motion. In other words, given the appropriate transform, genomic GC content can be seen to be just as likely to increase as to decrease. This is a scenario that can arise when DNA mismatch and repair enzymes are knocked out and the species is subjected to reduced selective pressures which is sometimes recreated in laboratory settings such as the long term evolutionary experiment (LTEE)^8^. The random perturbations of AT/GC mutation rates are represented here as Gaussian white noise multiplied by an unspecified deterministic measurable function *c*(*t*). Together with the Gaussian white noise term $W_t(\omega )$, the function *c*(*t*) allows for modeling of environmental influences and species-specific traits perturbing the AT/GC mutation rates over time *t*. Since Eq. (4) is a stochastic differential equation, with some restrictions it can be transformed into a Brownian motion if the SNP GC content of the organism is to be modelled as being just as likely to increase as to decrease. Such a model may be suitable for organisms in laboratory settings where selective pressures are absent with DNA mismatch and repair enzymes knocked out. If it is assumed that $t\in [0,T]$ for a fixed time *T*, then:

12 $$\begin{aligned} dF_t (\omega ) =((a-b)F_t (\omega ) + b)dt+c(t)dB_t (\omega ). \end{aligned}$$

To see that the Girsanov theorem applies to $F_t(\omega )$ recall that for $Y_t (\omega ) = e^{(-(a-b)t)} F_t (\omega )$:

13 $$\begin{aligned} dY_t = be^{(-(a-b)t)}dt+c(t)e^{(-(a-b)t)}dB_t. \end{aligned}$$

Since $F_t (\omega )$ is a semi-martingale, the Girsanov II theorem (p. 167^24^) can be used. Let

$$\begin{aligned} c(t)e^{(-(a-b)t)}u(t,\omega )=be^{(-(a-b)t)}-\phi (t,\omega ), \end{aligned}$$

and thus:

$$\begin{aligned} u(t,\omega )=c^{-1}(t)\bigg (b-\phi (t,\omega )e^{(a-b)t}\bigg ). \end{aligned}$$

$\phi (t,\omega )$ can then be set to zero so that:

$$\begin{aligned} u(t,\omega )=c^{-1}(t)b, \end{aligned}$$

which means that *c*(*t*) in Eq. (4) is required to have an inverse function $c^{-1}(t)$. Let

$$\begin{aligned} M_t = \exp {\Big (-\int _0 ^t u(s,\omega )dB_s - \frac{1}{2}\int _0 ^t u^2 (s,\omega )ds\Big )}, \end{aligned}$$

and set

$$\begin{aligned} dQ(\omega ) = M_t (\omega )dP(\omega ), \end{aligned}$$

with respect to the filtration $\mathcal {F}_t$, assume that the Novikov condition (see p. 165^24^) holds:

$$\begin{aligned} E\bigg [\exp {\Big (\frac{1}{2} \int _0 ^t u^2(s,\omega )ds\Big )}\bigg ]<\infty , \end{aligned}$$

so that the Radon–Nikodym derivative $M_t$ is a martingale. Then *Q* is a probability measure with respect to the filtration $\mathcal {F}_t$ and

$$\begin{aligned} \tilde{B_t}(\omega )=\int _0 ^t u(s,\omega )ds+B_t (\omega ), \end{aligned}$$

is a Brownian motion with regards to the measure *Q* and so is $Y_t$, according to the Girsanov theorem:

$$\begin{aligned} dY_t (\omega ) = c(t)d\tilde{B_t}(\omega ). \end{aligned}$$

Since $Y_t(\omega )=e^{(-(a-b)t)} F_t (\omega )$ it is clear that $F_t(\omega )$ is also a Brownian motion with respect to *Q*. Hence, given the right transform $F_t (\omega )$ is just as likely to increase as to decrease. If $F_t (\omega )$ is considered to represent a culture in an LTEE-type experiment^8,26,27^, where mismatch and repair genes are knocked out with selective pressures assumed to be at a minimum, and a transform, as discussed above, can be justified, the function *c*(*t*) must have an inverse, i.e. *c*(*t*) must be either monotonically increasing or decreasing. In evolutionary terms, an increasing *c*(*t*) could potentially be interpreted as extinction, for reasons described above, while a decreasing *c*(*t*) may be reflective of a bottleneck event.

### Calculation and presentation of the models

The figures based on the models described above were made with Julia version 1.6.1^28^. The “Differential Equations” library was used to compute the differential equations numerically. The ordinary differential equation models (ODEs) were estimated with the “Tsit5” algorithm (the fifth order adaptive time stepping method) while the stochastic differential equation based models were calculated with the EM method (Euler–Maruyama). All figures were created with Julia and the “Plot” library.

## Results and discussion

### The main model

As mentioned above, the present work is based on a previous study^11^ where the aim was to model the evolution of genomic GC content, as a consequence of AT/GC mutation rates, in microbial symbionts over time *t*. The present work however is not limited to the genomes of microbial symbionts, which tend to live in a stable relationship with their hosts, but to all asexually reproducing organisms regardless of which kingdom they belong to, including virus. It is still assumed that the genomes of the organisms considered comply with Chargaff’s parity laws^4^. In particular, it is assumed that genomic %G is approximately equal to %C and that %A is similar to %T on each strand in the genomes of the organisms considered. Although Chargaff’s parity laws were stated for most organisms with double stranded DNA genomes they also apply to many viruses with single stranded RNA genomes^6^. Indeed, the pathogen responsible for the currently ongoing Covid-19 pandemic, SARS-CoV-2, has a single strand, positive sense, RNA genome that obeys Chargaff’s parity rule with approximately 38% GC^29,30^. Nevertheless, increasing the generality of the previous model to the genomes of all asexually reproducing organisms implies that the assumption of constantly scaled random perturbing AT/GC mutation rates, which could be justified in a stable host-symbiont relationship, is no longer tenable. It is now therefore assumed that the random perturbations to AT/GC mutation rates vary according to a Gaussian white noise multiplied by a measurable function *c*(*t*) with respect to time *t* as previously deduced, Eq. (7):

$$\begin{aligned} \frac{dF_t (\omega )}{dt} =aF_t (\omega ) + b(1-F_t (\omega ))+c(t)W_t (\omega ). \end{aligned}$$

If restrictions on the function *c*(*t*) can be justified in a modeling setting Eq. (7) can be applicable to the Girsanov transform^24^ making the whole model $F_t$ a Brownian motion in it self. This implies that $F_t$ is just as likely to increase as to decrease with respect to the measure resulting from the Girsanov transform. In other words, it can then be guaranteed that $F_t(\omega )$ is an unbiased Brownian motion relative to a measure $Q(\omega )$. SNP GC content then follows a completely random path $\omega \in \Omega$ according to the law of Brownian motion. Due to influences from positive- and negative selection and mismatch and repair systems this is typically not observed outside laboratories^8^.

**Figure 1:**
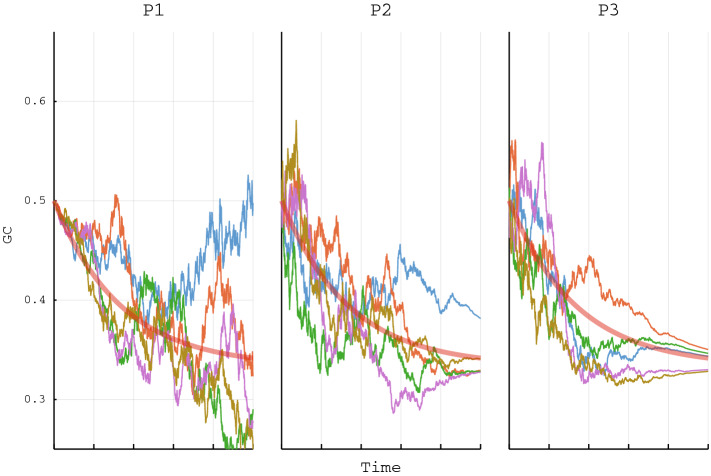
The figure demonstrates three different (P1–P3) evolutionary scenarios, each realized 5 times, for the model describing the evolution of genomic GC content (vertical axis) of a species over time *t* (horizontal axis) as a consequence of AT/GC mutation rates. The thick red line represents the deterministic model without the Brownian motion term. All parameters are the same for all scenarios (i.e. $a = -\,2$, $b = 1$, $F_0 = 0.5$ and $T = 1$) except for the function *c*(*t*) that determines the influence of the random perturbations on the AT/GC mutation rates. Panel P1: $c(t)=\frac{1}{2}\sqrt{t}$, Panel P2: $c(t)=\frac{1}{2}(T-t)$, Panel P3: $c(t)=\frac{1}{2}(T-t)^2$.

### The evolutionary dynamics of *c*(*t*)

AT/GC mutation rates are allowed to have random perturbation varying according to a function *c*(*t*) with respect to time *t*, i.e. $\alpha = a + c(t)W_t (\omega )$ and $\beta =b+c(t)W_t (\omega )$ where $W_t (\omega )$ is a Gaussian white noise with respect to every trajectory $\omega \in \Omega$. This has implications for the resulting Brownian motion term Eq. (10):

$$\begin{aligned} \int _0 ^t c(s)e^{(a-b)(t-s)}dB_s. \end{aligned}$$

Indeed, an increasing *c*(*t*) results in greater variance for $F_t(\omega )$ as $s\rightarrow t$ while a decreasing *c*(*t*) results in reduced variance. Figure 1 demonstrates $F_t(\omega )$ for different *c*(*t*) functions. Increased variance of $F_t(\omega )$ can be interpreted as increasing the intrinsic genomic base composition variation. However, increased genomic base composition variance (i.e. genomic GC content variance) could also describe accumulation of fitness decreasing, and even deleterious, mutations^15,31^. Hence, increased variance with regards to perturbation of AT- and GC mutation rates highlights the trade-off between more genetic diversity versus an increased chance of accumulating fitness decreasing and deleterious mutations and subsequent extinction. For decreasing *c*(*t*), variation in base composition is reduced, something that is likely to happen in a scenario of increasing purifying selection^15,32^. Organisms living in large populations^33^ or highly adapted to their environments^15^ would arguably be expected to have lower *c*(*t*) values than organisms subjected to more diverse and changing environments. It has previously been shown that nucleotide diversity in microbes increases with AT content^15^. That is, GC rich bacteria tend to have a more homogeneous nucleotide composition while AT rich bacteria have genomes comparably more heterogeneous in terms of nucleotide composition^34^. The underlying reasons for this decreasing nucleotide diversity gradient, from AT rich to GC rich genomes, is not known but years of research has shed some light on the issue. For instance, GC rich bacteria are mostly found in soil, with excess nitrogen^32^. Soil bacteria have large genomes and are often capable of metabolising a wide range of compounds^13^. AT-rich bacteria, on the other hand, are often symbionts^35^ and pathogens^36^ with reduced genome sizes. As mutations in prokaryotes are universally biased towards increased AT content^7^, loss of proof reading enzymes as well as DNA mismatch and repair genes, both hallmarks of relaxed selection, could be driving the greater genetic diversity found in these bacteria^37^. It has also been shown that the energetics of stacked A/T and G/C nucleotides is important in establishing genomic base composition^5^. Between-strand binding of A to T requires only two hydrogen bonds as compared to the three hydrogen bonds required for G to C bindings^1^. Guanine and cytosine also require the availability of more nitrogen^38^. Within eukaryotic genomes, GC content vary considerably more than within prokaryotic genomes^6^. The genomes of most eukaryotes have a substantially lower fraction of gene-coding DNA allowing for greater variation in GC content implying an increasing *c*(*t*)^6^. Viral genomes often mimic their hosts with respect to GC content and since their genomes are small, with a high fraction of coding genes, genomic GC content is typically far more stable than that observed for eukaryotic genomes and often comparable to prokaryotes^6^. A lower *c*(*t*) is therefore likely more suitable for both virus and prokaryotes as compared to most eukaryotes.

### Evolutionary implications of the model

The selective pressures a species is subjected to can, to some extent, be modeled by the measurable function *c*(*t*). In such cases, *c*(*t*) should be as close to zero as possible to avoid excessive hitch-hiking of fitness decreasing or deleterious mutations^31^. However, it will likely require several trade-offs for species’ to reduce the random variation of AT- and GC mutation rates representative of low *c*(*t*) values; considerable resources must likely be divested to several genomic processes to assure that fitness decreasing and deleterious mutations are purged. Moreover, a changing environment may require species’ to adapt rapidly implying the availability of an increased number of genotypes. A greater number of genetic variants require that the mutation rates reach a level that maximizes chance for survival of the species^39^. At the same time, if mutation rates increase to such an extent that deleterious mutations cannot be avoided or purged by selection the evolutionary process of Muller’s ratchet will ensue^22^.

Whether mutations increase or decrease, genomic GC content depends on the environment and the selective pressures operating on the species’ genomes. Some environments could favour energetically affordable A/T nucleotides while others might require the more costly G/C nucleotides^5^. In addition, phylogeny will also influence the selection of A/T or G/C nucleotides^13^ as a consequence of the mismatch repair system and/or proof reading enzymes^8,15,40^.

While there are many examples of microbial genomes becoming more AT rich^32^ there are so few examples of genomes becoming more GC rich that it was recently suggested that it may not happen at all^16^. Some examples have however been observed in the microbial world although the increase is minuscule^14,15,41^. It is not completely resolved whether the few examples of microbial genomes becoming more GC rich is tied to recombination^42^, which seems to be the case for recombining eukaryotes, or selection^6,14,17^.

Muller’s ratchet can, with respect to the model discussed here, be interpreted, in certain circumstances, as when the Brownian motion term completely overwhelm the AT/GC mutations rate terms in Eq. (3). This can be seen in Fig. 1 panels P1–P2, while panel P3 is an example of *c*(*t*) diminishing it’s influence with time, which can be interpreted as a population being subjected to increasing selective pressures. It is interesting to note that allowing for random perturbation of AT/GC mutation rates introduces a term that will unequivocally lead to greater variability of genomic base composition (see Eq. (6)); the larger the perturbations the greater the impact of the Brownian motion term on genomic variability as can be seen in Fig. 1. Put differently, according to the presented model genomic base composition can be modified by varying the random perturbations of the AT/GC mutation rates. It is therefore not unlikely that this is one of the reasons that mutation rates are occasionally found to be remarkably stable over a diverse set of organisms and, in particular, negatively associated with population- and genome size in single cell organisms^25,33,43^.

Assuming that Eq. (7) is a Brownian motion, in the sense that it is just as likely that GC content will increase as it will decrease, it is required by the Girsanov theorem, as seen in the Methods section, that *c*(*t*) has an inverse function meaning that *c*(*t*) must either be increasing or decreasing. Depending on the context, an increasing *c*(*t*) can be interpreted as the process of Muller’s ratchet, and thus subsequent extinction, due to an implicit accumulation of fitness decreasing and deleterious mutations. In other words, if it can be argued that *c*(*t*) should not be decreasing (i.e. resources are finite), increasing random perturbations affecting AT- and GC mutation rates could lead to genome decay. Interestingly, this has been demonstrated in a laboratory experiment and described in a recent study based on the LTEE^8^. Nevertheless, it should be emphasized that the mathematical model does not intrinsically include the concept of fitness and therefore all such explanations are necessarily interpretations.

### Evolution of genomic GC content and the Luria–Delbrück mutation model

By setting $\beta =0$ in

$$\begin{aligned} \frac{dF_t (\omega )}{dt} = \alpha F_t (\omega ) + \beta (1-F_t (\omega )). \end{aligned}$$

Equation (3) can be interpreted as a simple model for stochastic population growth (p. 65^24^):

$$\begin{aligned} \frac{dP_t (\omega )}{dt} = \pi P_t (\omega ), \end{aligned}$$

where $\pi = k + pW_t(\omega )$, *k* is the growth parameter to be estimated. *p* is considered to be a constant in this setting so that an analytic solution is possible. By using the Itô formula, along the lines described in the Supplementary Appendix and Ref.^24^, to solve for $P_t(\omega )$, and, subsequently, multiplying with a parameter $\mu$, taken to be a parameter designating mutations per unit time, a simple model for calculating the number of mutations in a population is obtained. This is an stochastic Itô calculus version of the model presented in the classical Luria–Delbrück fluctuation experiment^23^ (see Supplementary Appendix), i.e.

$$\begin{aligned} M_t(\omega )=P_t(\omega ) \mu = P_0\mu \exp {\bigg (\bigg (k-\frac{p^2}{2}\bigg )t + p B_t(\omega )\bigg )}. \end{aligned}$$

**Figure 2:**
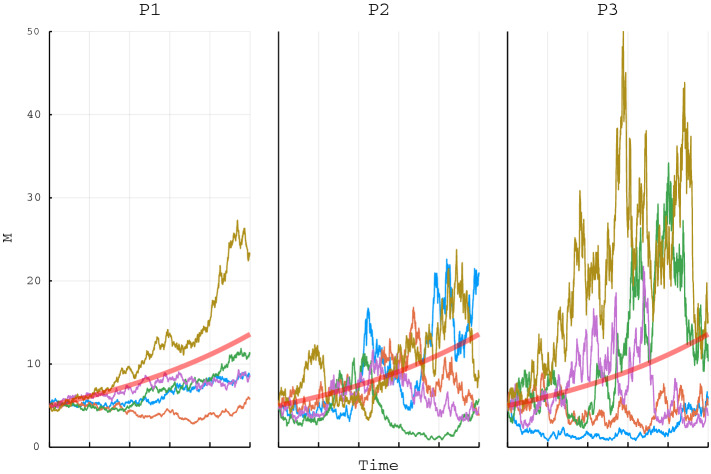
The figure demonstrates three different (P1–P3) scenarios for the stochastic Luria–Delbrück model, each realised 5 times, showing the mean number of mutations $M_t(\omega )$ (vertical axis) in a population at time *t* (horizontal axis). All parameters are the same for all scenarios ($P_0 =1$, $\mu = 5$, $k = 1$ and $T=1$) except for *p* that determines the influence of the random term. Panel P1: $p=\frac{1}{2}$, Panel P2: $p=\sqrt{2}$, Panel P3: $p=2$. The red line designates the Luria–Delbrück model without the Brownian motion term.

Hence, in this simple model the number of mutations in the study population is equal to the population size $P_t(\omega )$ multiplied with the mutation rate $\mu$. It can be seen from the above equations, as well as in Fig. 2, that as *k* approaches $\frac{p^2 }{2}$ a randomly fluctuating population will increasingly influence mutation rates. In other words, as the size of the population, as well as the growth rate, declines stochastic effects become more dominant^44^ resulting not only in increased genetic variation (see Fig. 2) but also the danger of decay due to accumulation of fitness decreasing and deleterious mutations from genetic drift^33^.

$M_t(\omega )$ represents the total number of mutations in a population $P_t(\omega )$, typically represented by mutations in one genome. If we assume that also $M_t(\omega )$ complies with Chargaff’s parity rules (see^19^) we can write:

$$\begin{aligned} M_t(\omega ) = M_t ^{AT} (\omega ) + M_t ^{GC} (\omega ), \end{aligned}$$

where $M_t ^{AT} (\omega )$ and $M_t ^{GC} (\omega )$ represents the number of A+T and G+C mutations, respectively. Equation (3) gives the fraction of GC mutations at time *t* and can be interpreted as the GC content of SNPs at that time. By multiplying Eq. (3) with the genome size *g* the number of GC mutations is given:

$$\begin{aligned} g \cdot \Big \vert \frac{dF_t (\omega )}{dt} = \alpha F_t (\omega ) + \beta (1-F_t (\omega )). \end{aligned}$$

However, $g \frac{dF_t (\omega )}{dt}$ will only be equal to $M_t (\omega )$ if $M_t (\omega ) = M_t ^{GC} (\omega )$. If $M_t (\omega ) = M_t ^{AT} (\omega )$, $g \frac{dF_t (\omega )}{dt}$ will be negative which is impossible to reconcile with the fact that $M_t(\omega )\ge 0$. But since the right hand side of Eq. (3), multiplied with *g*, can be written as:

14 $$\begin{aligned} g \cdot \Big \vert \alpha F_t (\omega ) + \beta (1-F_t (\omega )). \end{aligned}$$

It is clear from Eq. (14) that $M_t ^{GC} (\omega )$ and $M_t ^{AT} (\omega )$ respectively correspond to $\bigg |g\alpha F_t (\omega )\bigg |$ and $\bigg |g\beta (1-F_t (\omega ))\bigg |$. Therefore,

$$\begin{aligned} M_t(\omega ) = M_t ^{AT} (\omega ) + M_t ^{GC} (\omega ) = g\cdot \bigg (\bigg |\alpha F_t (\omega )\bigg | + \bigg |\beta (1-F_t (\omega ))\bigg |\bigg ), \end{aligned}$$

which is the number of mutations at time *t*.

A model for mutation accumulation can be written as the Lebesgue integral:

$$\begin{aligned} \int _0 ^t M_s(\omega ) ds, \end{aligned}$$

or:

$$\begin{aligned} \int _0 ^t g\cdot \bigg (\bigg |\alpha F_s (\omega )\bigg | + \bigg |\beta (1-F_s (\omega ))\bigg |\bigg ) ds. \end{aligned}$$

## Conclusions

The presented work has been concerned with modelling the evolution of genomic GC content, as a consequence of AT/GC mutation rates, in asexually reproducing organisms subject to Chargaff’s parity laws^4^. It is an extension of a previous study modelling genomic GC content in microbial symbionts allowing for random perturbations of AT- and GC mutation rates by the use of Itô calculus^24^. In that study^11^, it was shown that a symbiont’s life course could be determined when it entered into a relationship with it’s host. The present model does not allow for such a conclusion in general as the organisms modeled can both be diverse and live in very different environments. The evolution of the genomic GC content of these organisms can thus be better represented as a function *c*(*t*), regulating the influence of the random perturbations on AT/GC mutation rates, as opposed to a constant for microbial symbionts. An increasing *c*(*t*) will reflect greater base composition diversity but also implicit genetic hitch-hiking of fitness decreasing and deleterious mutations^31^. Processes described by a low *c*(*t*) will likely reduce genetic variation but require the divestment of increasing resources to mismatch and repair systems. Interestingly, Eq. (7) implies that increasing the variability of the random perturbations of the AT- and GC mutation rates impacts genomic GC content through the Brownian motion term.

Laboratory based evolutionary experiments^8,26,27^ often arrange conditions so that the selective forces subjected to the species’ studied are as low as possible. Furthermore, recombination related genes as well as mutation repair enzymes are often knocked out^8^ reducing bias considerably with regards to AT $\rightarrow$ GC and GC $\rightarrow$ AT mutation rates. The model presented suggests that if genomic GC content is just as likely to increase as to decrease *c*(*t*) must either be monotonically increasing- or decreasing due to constraints resulting from the Girsanov transform. An interpretation of this is that if not resources are limitless a constantly increasing *c*(*t*) will eventually represent genomic disintegration, as described by Muller’s ratchet^22^, something that has been demonstrated experimentally^8^. Furthermore, it is shown that there exists an intimate relationship with the model presented here and the classical Luria–Delbrück model for general mutations^23^. Indeed, disregarding AT mutation rates by setting $\beta =0$ in Eq. (3) gives an identical model to simple stochastic population growth $P_t(\omega )$^24^ which, when multiplied with a mutation rate $\mu$, gives a stochastic Luria–Delbrück model for the number of mutations $M_t(\omega )$. After some re-arrangements, it is shown above that Eq. (3) is related to $M_t(\omega )$.

Finally, Itô calculus facilitates modeling of phenomena often found in complex systems such as financial markets^45^. An additional consequence of the present study is that Itô calculus also models biological phenomena seamlessly thanks to the ability of handling random events in differential equations.

## Supplementary Information

Supplementary Information.

## Data Availability

The datasets used and/or analysed during the current study available from the corresponding author on reasonable request.
