# Supplementary material for: A simple stochastic model describing the evolution of genomic GC content in asexually reproducing organisms
Source: Sci Rep. 2022 Nov 3;12:18569. doi: 10.1038/s41598-022-21709-z (PMC9631610; doi:10.1038/s41598-022-21709-z)
Supplement: Supplementary file 1 — Supplementary Information. [file 41598_2022_21709_MOESM1_ESM.pdf]

# Appendix: A simple stochastic model describing the evolution of genomic GC content in asexually reproducing organisms

Jon Bohlin

August 18, 2022

This Appendix provides explanations for relevant formulas discussed in the main article.

## 1 Formulation of the deterministic genomic GC content equation

Assume that $F_{GC}(t)$ give the genomic GC content at time $t$. It is assumed that the change in genomic GC content during a small interval $\Delta t$ can then be written as:

$$F_{GC}(t + \Delta t) - F_{GC}(t) = \alpha \Delta t F_{GC}(t) + \beta \Delta t (1 - F_{GC}(t)) \tag{1}$$

Let $0 < F_{GC}(t) < 1$ so that $1 - F_{GC}(t)$ is AT content. Eq.(1) can then be interpreted as the change in genomic GC content during time interval $\Delta t$. The model assumes that the sum of the $AT \to GC$ and $GC \to AT$ mutation rates, meaning all possible combinations of $A/T \to G/C$ and $G/C \to A/T$ mutations, respectively, equals the GC content of variable sites/single nucleotide polymorphisms (SNPs). By dividing eq.(1) with $\Delta t$, we get:

$$\frac{F_{GC}(t + \Delta t) - F_{GC}(t)}{\Delta t} = \alpha F_{GC}(t) + \beta(1 - F_{GC}(t))$$

by letting $\Delta t \to 0$ we get a first order differential equation:

$$\frac{dF_{GC}(t)}{dt} = \alpha F_{GC}(t) + \beta(1 - F_{GC}(t)) \tag{2}$$

The right-hand side can be re-written as

$$\frac{dF_{GC}(t)}{dt} = (\alpha - \beta) F_{GC}(t) + \beta$$

By rearranging slightly, it can be seen that

$$\frac{dF_{GC}(t)}{dt} = \beta\Big(\frac{(\alpha - \beta)}{\beta} F_{GC}(t) + 1\Big)$$

and that

$$\frac{dF_{GC}(t)}{\Big(\frac{(\alpha-\beta)}{\beta} F_{GC}(t) + 1\Big)} = \beta dt$$

which can be solved in the usual way using logarithms to give the formula:

$$F_{GC}(t) = \frac{\beta}{\alpha - \beta}(Ce^{(\alpha-\beta)t} - 1) \tag{3}$$

The mutation rate parameters $\alpha$ and $\beta$ can be resolved using empirical data (see [1, 2] for how this can be carried out) while the constant $C$ is determined according to the model specification (*i.e* initial conditions). This model was derived from a model where the GC content of genomic variable

sites/SNPs was a function of core genome GC content and assumed to be the summation of AT/GC mutation rates [1, 2], *i.e.* :

$$F_{GC}(x + \Delta x) = F_{GC}(x) + \alpha \Delta x F_{GC}(x) + \beta \Delta x (1 - F_{GC}(x))$$

In this setting, $F_{GC}(x)$ represents genomic GC content as a function of $x$, core genome GC content, as opposed to time in the above formulation. $\Delta x$ represents the change in core genome GC content. More specifically, genomic GC content was assumed to be a function of core genome GC content calculated from $\alpha$ and $\beta$ ($AT \to GC$ and $GC \to AT$ mutation rates, respectively):

$$F_{GC}(x) = \frac{\beta}{\alpha - \beta}(Ce^{(\alpha-\beta)x} - 1)$$

Although the formulations are identical and give the same solution for $x = t$ (eq.(3)), their interpretation is quite different.

### 1.1 The classical Luria-Delbrück equations

The original Luria-Delbrück model [3] assumes that the number of mutations in a population can be formulated as a mutation rate $\mu$ multiplied by population size $P$ at time $t$ from a simple growth model:

$$\frac{dP}{dt} = kP \tag{4}$$

where $k$ is taken to be the growth rate. Since

$$\frac{dP}{P} = kdt$$

it is straight-forward to show, by integrating both sides, that the solution to eq.(4) is $P(t) = P_0 \exp(kt)$, with $P_0$ as the initial population size. This implies that the mean number of mutations at time $t$ can be given by the following formula:

$$m(t) = \mu P(t) = \mu P_0 \exp(kt) \tag{5}$$

### 1.2 The simple stochastic growth model

Derivation of the simple stochastic growth model:

$$\frac{dP_t(\omega)}{dt} = \pi P_t(\omega)$$

for the stochastic Luria-Delbrück model can be performed as follows. Let $\pi = k + pW_t(\omega)$, which gives:

$$\frac{dP_t(\omega)}{dt} = (k + pW_t(\omega))P_t(\omega)$$

and therefore

$$\frac{dP_t}{P_t} = kdt + pdB_t$$

This can now be integrated:

$$\int_0^t \frac{dP_s}{P_s} = kt + pB_t$$

Let $g(t, x) = \ln x$ and use Itô's formula:

$$d(\ln P_t) = \frac{1}{P_t} dP_t + \frac{1}{2}\Big(-\frac{1}{P_t^2}\Big)(dP_t)^2$$

Since

$$dP_t = kP_t dt + pP_t dB_t$$

it follows that:

$$\begin{aligned} d(\ln P_t) &= \frac{dP_t}{P_t} - \frac{1}{2P_t^2} p^2 P_t^2 dt \\ &= \frac{dP_t}{P_t} - \frac{p^2}{2} dt \end{aligned}$$

plugging:

$$\frac{dP_t}{P_t} = kdt + pdB_t$$

into:

$$d(\ln P_t) = \frac{dP_t}{P_t} - \frac{p^2}{2} dt$$

gives:

$$d(\ln P_t) = kdt + pdB_t - \frac{p^2}{2} dt = (k - \frac{p^2}{2})dt + pdB_t$$

After taking limits, this can now be written as

$$\ln \frac{P_t}{P_0} = (k - \frac{p^2}{2})t + pB_t$$

so that:

$$P_t = P_0 \exp((k - \frac{1}{2} p^2)t + pB_t)$$

# References


[1] Bohlin, J., Eldholm, V., Brynildsrud, O., Petterson, J. H. O., & Alfsnes, K. *Modeling of the GC content of the substituted bases in bacterial core genomes.* BMC genomics, **19(1)** 589 (2018)

[2] Bohlin, J., Rose, B., & Petterson, J. H. O. *Estimation of AT and GC content distributions of nucleotide substitution rates in bacterial core genomes.* Big Data Analytics, **4(1)** 1-11 (2019)

[3] Luria, S. E., & Delbrück, M. *Mutations of bacteria from virus sensitivity to virus resistance.* Genetics, **28(6)** 491 (1943)

[4] Øksendal, B. *Stochastic differential equations.* Springer, Berlin, Heidelberg (2003)
